# Deep sequencing of large library selections allows computational discovery of diverse sets of zinc fingers that bind common targets

**DOI:** 10.1093/nar/gkt1034

**Published:** 2013-11-07

**Authors:** Anton V. Persikov, Elizabeth F. Rowland, Benjamin L. Oakes, Mona Singh, Marcus B. Noyes

**Affiliations:** ^1^The Lewis-Sigler Institute for Integrative Genomics, Princeton University, Princeton, NJ 08544, USA, ^2^Department of Computer Science, Princeton University, Princeton, NJ 08544, USA and ^3^Department of Molecular Biology, Princeton University, Princeton, NJ 08544, USA

## Abstract

The Cys_2_His_2_ zinc finger (ZF) is the most frequently found sequence-specific DNA-binding domain in eukaryotic proteins. The ZF’s modular protein–DNA interface has also served as a platform for genome engineering applications. Despite decades of intense study, a predictive understanding of the DNA-binding specificities of either natural or engineered ZF domains remains elusive. To help fill this gap, we developed an integrated experimental-computational approach to enrich and recover distinct groups of ZFs that bind common targets. To showcase the power of our approach, we built several large ZF libraries and demonstrated their excellent diversity. As proof of principle, we used one of these ZF libraries to select and recover thousands of ZFs that bind several 3-nt targets of interest. We were then able to computationally cluster these recovered ZFs to reveal several distinct classes of proteins, all recovered from a single selection, to bind the same target. Finally, for each target studied, we confirmed that one or more representative ZFs yield the desired specificity. In sum, the described approach enables comprehensive large-scale selection and characterization of ZF specificities and should be a great aid in furthering our understanding of the ZF domain.

## INTRODUCTION

**Figure 1.**
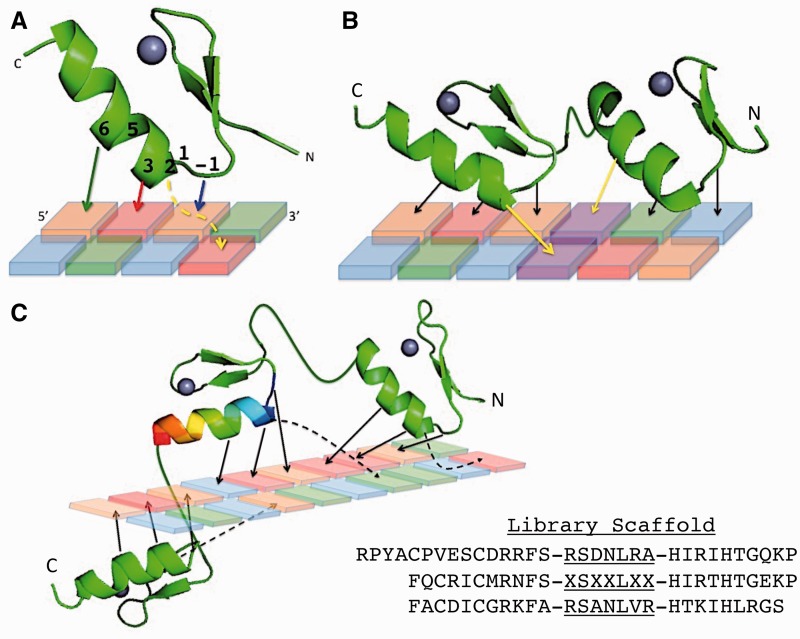
Zinc finger–DNA interactions. (**A**) A single zinc finger is shown. The four ‘canonical’ (5) contacts are noted with colored stick arrows between the specified positions of the recognition helix. Positions -1–6 of the recognition helix are labeled. The cross-strand canonical contact between position 2 of the helix and the base immediately 3′ to the core 3 bp target is signified by a yellow dashed line. (**B**) Two zinc fingers are shown that highlight how zinc finger targets can overlap. The contacts from the N-terminal and C-terminal fingers that both specify the same purple base pair are noted with yellow arrows. (**C**) A three-fingered protein is shown with a 10-bp target. All four canonical contacts from each finger are shown with arrows and cross-strand contacts with dashed arrows. The 3D design is meant to highlight how the zinc fingers wrap around the DNA and bring contacts by distal fingers close together in 3D space. The helix of the central finger is shown as a rainbow to signify that it contains the randomized library positions of the ‘RA’ library processed in this manuscript. The scaffold sequence for the library is given, with the -1–6 positions of each helix underlined, and the randomized positions indicated by ‘X’. Zinc finger structures are modified from (6).

The Cys_2_His_2_ zinc finger (ZF) is the most common DNA-binding domain found in metazoans (1–3). Nearly 50% of the transcription factors (TFs) in the human genome are thought to use ZFs to recognize their targets (2–4), yet characterization of their DNA-binding specificities has proven difficult. Within these factors, a single ZF domain binds 3–4 bases of DNA, whereas proteins often contain arrays of multiple adjacent ZFs (Figure 1). Though numerous variations are possible, each ZF within a canonical array (5) binds DNA in ‘modules’ of 4 bp that overlap in a single nucleotide position (Figure 1b). Adjacent ZFs in these canonical arrays may have complementary or conflicting nucleotide preferences in the overlap position.

Natural ZF proteins bind a wide range of DNA sites, and their relatively modular interactions, as gleaned from structure, have been leveraged to engineer ZF proteins with desired specificities (6–12). In principle, individual ZF domains with known specificities can be assembled in tandem to bind larger sites that are a concatenation of the individual subsites. However, modular assembly of ZFs has a high failure rate when challenged to activate a cell-based reporter assay, perhaps due to conflicting preferences in overlap nucleotides, indicating that we do not fully understand the dependencies between neighboring fingers that influence functional assembly (13). It should be noted that some of these same fingers were characterized with an alternative method and the desired specificities were mostly revealed (14). Further, zinc fingers assembled to include 4–6 fingers have demonstrated much greater success that may imply that conflict at the overlap may influence affinity (15), but a disfavored pairing can ultimately be overcome when assembling longer ZF arrays. As a result, engineered ZFs are powerful tools for targeting auxiliary domains to genomic targets [reviewed in (16)]. ZF-nucleases and recombinases have enabled fine genome editing and have proven function in many model organisms (17–23). ZF activators can be engineered to regulate a target of interest or control networks (24–26). ZF fusions to methyltransferases have been used to methylate DNA or histones site specifically (27–29).

Given the importance of ZFs in cellular networks and their use in genome engineering applications, characterizing their sequence-specific DNA binding is of great interest. A wide range of experiments including Systematic Evolution of Ligands by Exponential Enrichment (SELEX), phage display, protein binding microarray and bacterial one-hybrid (B1H) selection have been used both to uncover the specificities of ZFs as well as to select new ZFs to bind DNA sites of interest (30–40). As a result, ZFs are perhaps the most well-characterized DNA-binding domain, with several existing computational approaches that use data from these experiments to predict ZF binding sites (41–45). Despite these successes, we have yet to obtain a predictive understanding of the ZF domain. It has also proven difficult to engineer ZFs to specifically bind every possible 3 bp binding site (46) or to assemble them as functional arrays (13). Further, two recent large-scale studies to characterize the binding specificities of naturally occurring ZF proteins from human and fruit fly failed to characterize 92 and 62% of these factors, respectively, whereas all other domains tested offered high success rates (3,47). A better understanding of the ZF domain is clearly necessary to uncover, engineer or predict the *in vivo* targets of these proteins.

To expand the knowledge base of possible ZF–DNA interfaces, we developed a high-throughput method for selecting ZFs with desired specificities that overcomes some of the limitations of current approaches. Existing methods for ZF selection such as phage display, B1H and two-hybrid and yeast one-hybrid systems (10,17,48–53) may recover primarily high-affinity zinc fingers as a small number of clones are usually used to find a consensus ZF amino acid profile that represents a given target specificity. Analysis of a small number of selected clones may bias the results toward the most likely high-affinity candidates at the expense of lower affinity and possibly more specific ZFs. Further, many previous approaches use incomplete coding schemes in their ZF libraries or fix residues on the recognition helix to limit the library size required to cover all possible variants (17,52,54–57); this may exclude unique combinations of amino acids required to specify a given target or bias the utility of these results to a limited application context. Finally, many previous approaches for selecting ZFs binding DNA targets have heavily relied upon high-affinity arginine-guanine contacts, and as a result, many of the publicly available ZFs are heavily biased toward GNN-binding fingers (50–52,58,59). These fingers may limit the transferability of the results as they may be primarily driven by the high-affinity contacts and not high overall specificity.

In contrast to previous approaches, we set out to build large diverse ZF libraries and to uncover a range of disparate ZFs. We developed and applied an experimental-computational pipeline to enrich and recover groups of distinct ZFs that bind a common target from a single selection. Our main contributions are as follows. First, we optimized a polymerase chain reaction (PCR)-based cassette mutagenesis method to build libraries of ZF proteins where up to six amino acid positions are varied, all 20 amino acids are possible and theoretical DNA library sizes of 3 × 10^7^ and 1 × 10^9^ are over-sampled by at least 5-fold. Second, we derived a simple analytical formula to calculate the expected diversity of a library and showed via high-throughput sequencing that the produced libraries offer levels of diversity that approach the theoretical maximum. Third, we used one of our ZF libraries with five varying amino acid positions in conjunction with the B1H system to select and deep sequence ZFs that bind several 3 bp targets of interest. Fourth, we developed an information-theoretic approach, based on the number of ways a protein sequence may be encoded that allowed us to uncover enriched ZFs (i.e. corresponding to ZFs binding the targets of interest) from large sequence pools that may contain considerable background. Fifth, because the sequencing depth of selected ZF pools resulted in thousands of enriched ZFs for each target, we clustered them to uncover distinct classes of similar amino acid profiles. Finally, for each target studied, we confirmed that one or more of the selected ZFs offer the desired specificity and further tested a subset of these to confirm that they can act as artificial TFs in yeast.

## MATERIALS AND METHODS

### Library oligonucleotide design

There were three critical regions considered for design of the library-encoding oligonucleotides: the annealing region, the library and the extension (see Supplementary Figure S1 for reference). The region that anneals to the template DNA was designed to have 65–70°C of annealing temperature. The randomized library region of the oligonucleotide was designed to introduce an NNS coding scheme for each codon to be randomized in the desired position of the ZF coding template. An NNS coding scheme results in 32 possible codons and codes for all possible amino acids plus one stop codon. The five prime extension of the oligo contains a restriction enzyme target as it was used here to capture the library PCR product by digestion and ligation with an extended piece of DNA. Template sequences are listed in the Supplementary Material.

### Library build procedure

The library build procedure was broken down into five basic steps: (i) library PCR first round; (ii) digestion/ligation capture; (iii) library PCR second round; (iv) digestion and ligation into the expression construct; and (v) transformation and expansion. We now describe each of these steps in more detail with an exhaustive description of the library build procedure provided in the Supplementary Materials.

#### Library PCR first round

In all, 48–96 PCR reactions were performed using the library-encoding oligonucleotide and an appropriate template. Each PCR reaction used Expand High Fidelity Plus (Roche, 04 743 733 001) and 15–20 cycles were carried out as listed in the Supplementary Methods. PCR reactions were pooled and recovered by gel electrophoresis and gel extraction.

#### Digestion/ligation capture

The recovered DNA is digested with the appropriate enzyme to assemble with the desired extension fragment (Supplementary Figure S1). Digests were recovered by gel electrophoresis and gel extraction. Finally, T4 DNA ligations were performed according to the manufacturer’s guidelines with a 1:1 molar equivalent of each fragment, separated on an agarose gel and the library-ligated band recovered.

#### Library PCR second round

The ligated library material was expanded by a second round of PCR using external primers, far removed from the library-coding region. PCR reactions were pooled and recovered by gel electrophoresis and gel extraction.

#### Digestion and ligation into expression construct

The expanded PCR material was digested with KpnI and XbaI according to the manufacturer’s guidelines and recovered by gel electrophoresis. T4 DNA ligase was used for large-scale library ligation. For each library build, 5–10 ligations were performed using a 5:1 molar ratio of insert to vector. Two micrograms of the Kpn1-XbaI-digested expression vector was used per 20 ul ligation. Ligations were held at 16°C for 12–14 h, followed by 20 min at 65°C. Ligations were ethanol precipitated and resuspended in 20 ul of Tris-buffer.

#### Transformation and expansion

A test transformation of 1 ul of the recovered ligation was used to determine the number of transformants each transformation will produce and help predict the number of transformants required for a given library build. In most cases, a single transformation results in 5 × 10^8^ to 1 × 10^9^ transformants. On electroporation, transformations were recovered in 1 l SOC [Super Optimal Broth (SOB) + 0.5% glucose]. For controls, an empty ligation using 1 ug of cut vector and no insert served as a control to determine the ligation fold over the background. Transformation of cells with no DNA was used as an expansion control. Cells were recovered for 45 min at 37°C with shaking. In all, 200 ul were recovered from each control and sample to quantify library build size (see later). Carbenicillin was added to each sample and control to select for the presence of the ligated plasmid. Cells were allowed to expand to an OD600 of 0.5–0.75 when using the expansion control as the blank (∼5–6 h). DNA was harvested and sequenced to confirm diversity.

Templates, library sequences and expression vectors are listed in the Supplementary Material.

### Calculating library build

To quantify library builds, the 200 ul recovered from each sample previously was diluted in 10-fold steps, plated on antibiotic containing media and grown at 37°C overnight. Once grown, the number of cells that took up plasmids from the transformation was calculated by counting colonies at the lowest dilution present and back calculating to quantify the size of the library build. We are able to confirm the fraction of cells that are transformed by library-ligated plasmid versus background by running a no insert control and making a comparison by which we typically find roughly 100-fold increase in the number of transformants per unit plated for the library insert relative to this control. The 100-fold over this background demonstrated here approximates that roughly 1% or less of our total library build is from background and 99% is from ligated library material. Further, the expression vectors cloned into all contain a non-coding cassette between the sites cloned into. Therefore, the small amount of background transformed in any selection will not contain a functional DNA-binding domain and will not survive the selection conditions.

### Library diversity analysis

The libraries were Illumina sequenced by the Princeton University High Throughput Sequencing facility. FastQC was used for preliminary data quality analysis and filtering, and all the sequences were processed further by custom Python scripts. First, all the low-quality sequences with mismatches observed either in regions of the amplified fragments that should have been constant codon positions or in the Illumina adapter regions were removed. The database of 15- or 18-bp-long nucleotide sequences corresponding to the 5 or 6 amino acid positions was collected.

To test the diversity of the designed libraries, we compared the number of total sequences processed with the number of unique sequences appearing in the library. For a sequence with *i* variable amino acid positions, encoded by the NNS codons, the total possible number of encoding DNA sequence variants could be computed as *N* = (4*4*2)^i^. Thus, for the libraries with six variable amino acid positions *N* = 1.07 × 10^9^, and for libraries with five variable amino acid *N* = 3.36 × 10^7^. In the ideal case, we would obtain a uniform distribution of the DNA variants in the library when sampled randomly, and we can compute the expected number of distinct (or unique) sequences *U* observed as a function of the total number sequenced *n* and the total number of possible variants *N* as:
(1)
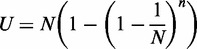

**Figure 2.**
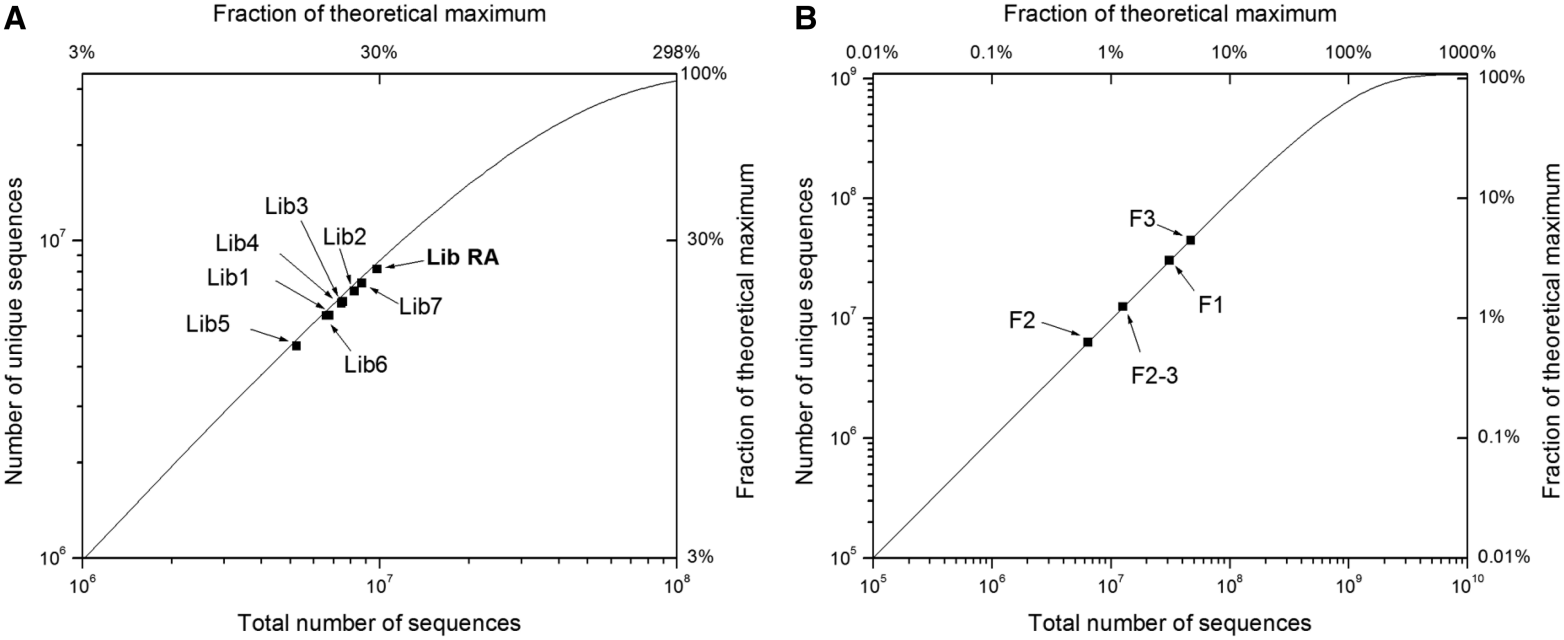
Library diversity determined by deep sequencing. (**A**) The number of distinct sequences recovered as a function of the number of total sequences is shown for eight libraries with five random amino acids, with the RA library processed in this article bolded. The theoretical line is a depiction of Equation (1) in the main text representing the number of distinct sequences one would expect as sequencing depth is varied from a uniformly distributed pool of sequences. The fraction of the theoretical maximum that is represented by the number of sequences processed is shown at the top. (**B**) The number of distinct sequences recovered as a function of the number of total sequences processed is shown for four libraries with six random amino acids.

When the number of studied sequences *n* is small (*n<<N*), the number of unique sequences observed *U* should be close to *n* (i.e. all the sequences are unique). However, at high sequencing depth (*n*→∞), *U* will be limited by the total number of possible variants *N*. To assess diversity, the number of unique sequences observed in filtered Illumina data was compared with the value predicted by Equation (1) at the same number of studied sequences (*n*). We also report sequence counts as a fraction of the theoretical maximum *N* (Figure 2).

### B1H zinc finger selection

Zinc finger selections were performed as previously described (17,35). Briefly, all ZF libraries were built in an expression vector that will express the ZF-omega fusion using a strong promoter. This vector offers the highest expression level of the previously described B1H vectors (34). One five amino acid library was chosen to test. This library is referred to as the ‘RA’ library because it expresses the helix RSDNL*RA* as the N-terminal finger (see Figures 1 and 2 and the supplement for details of the RA library). The ‘RA’ library vector was transformed along with one of the B1H reporter plasmids into the B1H selection strain. Each B1H reporter plasmid used per selection offered the target site of interest 10 bp upstream of the promoter that drives expression of the *HIS3* reporter gene (see Supplementary Material). After transformation with both plasmids, the cells were expanded, washed and a fraction plated in serial dilution and incubated at 37°C. The remaining cells were stored at 4°C overnight. Once grown, the serial dilutions were counted and roughly 1 × 10^8^ cells plated on selective media from the stored remainder of cells. Selections were always tested at a high (10 mM 3AT) and low (2.5 mM 3AT) stringency. This cell count represents a 3-fold over sampling of the five amino acid library investigated here. Cells were grown on the selection plates for 36–48 h at 37°C. Surviving cells were counted, pooled and prepped for Illumina sequencing.

### B1H binding site selection

ZF binding site selections were performed as previously described (34). Briefly, a vector that expresses a candidate ZF using the lacUV5 promoter was transformed along with a reporter plasmid library that contains a 28 bp region of randomized sequence upstream of the promoter that regulates the HIS3 reporter. This library has been previously described (34). Transformants were treated as previously, with the exception that 5 × 10^7^ cells were plated. Cells were grown on selective plates containing 5 or 20 mM 3AT, at 37°C for 36–48 h. Surviving cells were counted, pooled and prepped for Illumina sequencing.

### DNA prep for Illumina sequencing

A detailed description of the Illumina prep can be found in the Supplementary Methods. Briefly, colonies that survived the ZF and binding site selections as described previously were pooled and DNA harvested. The variable region of either the ZFs or the random binding site region of the reporter was amplified by PCR using the barcoded Illumina primers listed in Supplementary Table S1. The PCR reactions used Expand High Fidelity Plus (Roche, 04 743 733 001), 12 cycles, in a 96-well format, with one reaction representing each pool. The PCR products were recovered by gel electrophoresis and gel extraction. The concentration of each product was quantified (Thermo scientific, Nanodrop 2000c) and diluted to 10 nM. The barcoded products were combined and sequenced.

### Processing zinc finger selection data

Illumina sequencing and analysis were used to uncover amino acid sequences binding a desired DNA site. To uncover truly enriched ZFs from sequence pools that may contain significant background, we considered each way a protein could be encoded using NNS codons and computed the relative observation frequency *p_i_* of each such DNA sequence in the Illumina sequencing. The Shannon entropy (60), normalized by the number of possible ways to code the amino acid sequence using NNS codons, was used as a measure of the diversity with which that particular amino acid sequence was observed: 
(2)
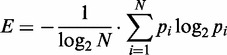

where *N* is the total number of DNA variants to encode the current amino acid sequence. Amino acid sequences with either normalized entropy ≥0.25 or that can only have a single possible encoding with NNS codons were retained for further analysis; the rest were removed, as the lack of diversity in their underlying coding sequences suggests that they may be artifacts. 

### Zinc finger cluster analysis

When similar combinations of amino acids result in binding the same DNA target, they can be clustered into ‘specificity groups’ of similar protein sequences that offer alternative binding strategies. Therefore, each selection experiment was described as a graph with amino acid sequences representing the nodes of the graph. The similarity between two sequences was computed using BLOSUM62 (61) and normalized to be between 0 and 1. Two nodes were connected with an edge if the similarity score between the two corresponding protein sequences exceeded 0.25. We used the SPICi program (62) with default parameters to identify clusters of 10 or more protein sequences. Finally, for each selection, we used the amino acid sequences that passed the entropy and clustering filters, along with the frequencies with which they occurred, to build a combined sequence logo (63).

### Processing binding site selection data

Illumina sequencing and analysis were used to uncover binding site preferences selected with candidate ZF proteins by B1H selection. FastQC was used for preliminary data quality analysis and filtering, and all the sequences were processed further by custom Python scripts. All low-quality sequences with mismatches observed in the constant adapter regions were removed. The database of 28 bp long nucleotide sequences containing the selection region was collected, keeping every sequence observed at least twice in the sequencing database. We observed that there were highly similar 28 bp sequences, and that these may cause difficulties for motif finding approaches. To find 10 bp enriched within these sequences, we greedily grouped any pair of 28 bp long sequences that differed by ≤2 nt positions. For each group of similar sequences, we chose the sequence with the highest counts as its representative sequence. We then used MEME to uncover the overrepresented 10 bp motifs (corresponding to the binding site length of three ZFs) that are found within the set of filtered sequences. The 10 bp matches to the motifs are extracted from the original set of sequences and represented by sequence logos using the log of the frequencies with which each sequence is observed.

### Zinc finger induction of GFP

*Yeast ZF strain and plasmid construction*: transformations were performed with a standard lithium acetate method. To construct all ZF-estrogen receptor-VP16 fusions (ZF-EVs), a previously described strain (25) was used that harbors the *URA3* gene between an integrated *ACT1* promoter and the estrogen receptor-VP16 coding sequence. Candidate ZFs were PCR amplified to include homology to the ACT1 promoter and the estrogen receptor, transformed into cells and selected via 5-FOA counter-selection of *URA3*. Single colonies were isolated and sequenced to verify the presence of the proper DNA-binding domain. The reporter plasmids used were modifications of a previously reported CEN plasmid containing the *URA3* selectable marker. ZF binding sites were cloned between the XbaI and NotI sites, placing them in a position with confirmed activity, of the promoter that drives green fluorescent protein (GFP) expression.

#### Induction

Three biological replicates were tested for each zinc finger binding site pair. Cells were grown in synthetic complete medium lacking uracil. Induction of ZF-EV activators by 100 nM β-estradiol (Tocris Biosciences, Ellisville, MO, USA) was performed in cells during log-phase growth (culture absorbance = 50–100 Klett units). Cells were recovered at 12 h post induction.

#### Flow cytometry

Approximately 10^7^ cells were harvested by centrifugation and subsequently washed and resuspended in Dulbecco’s phosphate-buffered saline (DPBS) + 0.1% Tween-20. Measurements of GFP fluorescence were performed with a BD LSRII Multi-Laser Analyzer with HTS (BD Biosciences, Sparks, MD, USA). Mean fluorescence values were determined from at least 50 000 cells. For each experiment, a positive control, Zif268 paired with its consensus target, and a negative control, Zif268 with an empty vector, were performed. The mean GFP fluorescence for each test zinc finger binding site pair was normalized to the positive control GFP output to control for variability between experiments.

## RESULTS

### Zinc finger library builds by PCR-driven cassette mutagenesis

We recognized that the routine production of large and diverse ZF libraries could greatly expand the number of known protein–DNA interactions mediated by this domain. To provide a straightforward method to produce such libraries, we optimized a PCR-based procedure to assemble and expand a library cassette that focuses on the steps that influence production and bias. The steps of the procedure are outlined in Supplementary Figure S1 and detailed in the Supplementary Methods. There were three foci at the core of the optimized method. First, a separation of 20°C between the designed annealing temperature of the library oligo and the temperature performed in the PCR reaction minimizes any amplification advantage that one library member may have over another (Supplementary Figure S2). Second, many reactions were carried out and pooled to dilute any bias that might occur in one reaction versus another. Finally, design of a restriction site at the 5′ end of the oligo allows for capture of full-length library fragments (Supplementary Figure S1). These fragments can be captured by digestion and ligation with another fragment of DNA. The ligated fragment can then be expanded with a second round of PCR using distal external primers that should expand the library fragment uniformly. The second expansion of the DNA allows for production of a great deal of material to be cloned into the expression vector of choice and maintain diversity from ligation to PCR expansion to the final library build (Supplementary Figure S3). This procedure has produced tens of micrograms of fully digested library cassette allowing for the routine production of 10^9^–10^10^ library builds (Supplementary Table S2). We have used this method to produce multiple ZF libraries where either 5 or 6 codons have been fully randomized using an NNS coding scheme (Figure 2). All library builds over-sampled the theoretical diversity by at least 5-fold. The sequence and randomized codons are detailed in the Supplementary Material.

### Measuring library diversity

We used Illumina sequencing along with theoretical calculations to characterize the diversity offered by our library building procedure. We compared the number of unique sequences recovered as a function of the total number sequenced with the number of unique sequences one would expect from a library with DNA sequences uniformly distributed (Figure 2). Eight libraries were built where five codons were randomized: positions -1, 2, 3, 5 and 6 of the recognition helix (Figures 1a and 2a) within the coding sequence for either the central or C-terminal ZF of a three-fingered protein. We recovered 5.3 to 9.8 × 10^6^ sequences for each of these libraries and find in each case ∼95% of the expected diversity (Figure 2a). In addition, we describe four libraries where six codons have been randomized: positions -1, 1, 2, 3, 5 and 6, resulting in a theoretical library size of 1.07 × 10^9^. We recovered 6 × 10^6^ to 4.5 × 10^7^ sequences from these libraries, which offered 98–99% of the expected diversity (Figure 2b); we note that these sequences represent only 0.6–4.2% of the theoretical maximum, and this level of diversity will likely be reduced when sampling a larger faction of the maximum. Though we are pleased with the high level of diversity across 12 libraries, it is worth noting that some of the small differences between sampled and expected diversity might be explained by bias in the synthesis of the library oligonucleotide. We find common bias at ‘N’ and ‘S’ positions for all libraries made with common library oligonucleotides (Supplementary Table S3). Libraries made with different oligonucleotides have their own separate common bias. These data imply that the slight bias observed may originate in the coding oligonucleotide and not the library building process.

### Computational pipeline of ZF selections

To test the functionality of a ZF library with the confirmed diversity offered earlier in the text, we focused on a single library where the central finger is randomized. This library consists of a three-fingered array that surrounds the randomized center finger with a GAG-binding C-terminal finger and a N-terminal finger that specifies 5′ (a/c)AG 3′. This library was chosen because the recognition helix of the N-terminal finger, RSDNL*RA*, provides flexibility in the specificity immediately 3′ to the bases the library finger should specify. This flexibility is presumably because of the residues at positions 5 and 6 of the helix and is therefore referred to as the ‘RA’ library. Also to note, the C-terminal GAG-binding finger places an alanine at position 2 of the recognition helix (RS*A*NLVR) so as to avoid cross-strand contacts that could influence the selection. A previous report demonstrates that an alanine at position 2 of an N-terminal finger has little influence on the 3′ base preference (64). ZFs were selected from the RA library to bind to all sequences included in the 5′ *n*AG 3′ and 5′ CA*n* 3′ sets using a B1H system (34). This system offers a sensitive assay of a specific protein–DNA interaction while providing a level of non-specific competition from the bacterial genome. These selections enriched for hundreds or thousands of candidate ZFs (Supplementary Table S4). Surviving colonies from each selection were pooled and processed for sequencing.

Illumina sequencing and analysis were used to uncover preferences in the amino acid sequences binding a desired DNA site. To restrict our analysis to colonies that survive as the result of DNA binding as opposed to some other background artifact, we reasoned that if a particular amino acid sequence can bind the target DNA site, and that sequence can be encoded in several ways, we should recover a diverse set of underlying DNA sequences, particularly because of the confirmed diversity of our libraries. In contrast, if an amino acid sequence is represented primarily by a single-coding DNA sequence, this protein sequence should be considered background and removed from further analysis. It is also possible that a small amount of true positives are lost because they do not fold properly in bacteria.

**Figure 3.**
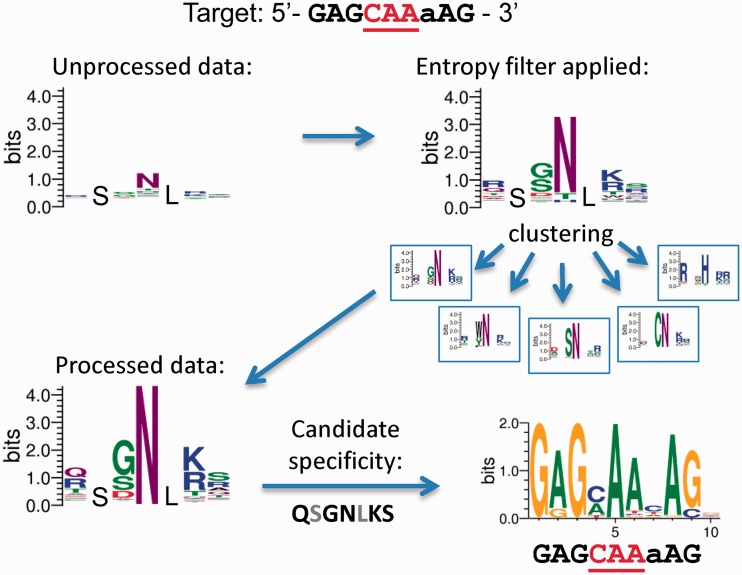
Overview of the computational pipeline for zinc finger selection analysis. The example details selection of the central finger in a three-fingered array to recognize the 10 bp target 5′-GAGCAA(a/c)AG-3′. The C-terminal and N-terminal constant fingers use the RSANLVR and RSDNL*RA* helices, which bind GAG and (a/c)AG, respectively. Five amino acid positions (-1, 2, 3, 5 and 6) were randomized with a constant serine at position 1 and leucine at position 4. An unprocessed data sequence logo reflects the raw counts of recovered sequences at five variable positions (top left). Next, after the entropy-based diversity filter is applied, the amino acid enrichment is shown as a sequence logo. Zinc fingers with similar amino acid profiles are clustered and the highly populated cluster used to determine a candidate protein to test specificity (QSGNLKS sequence for the finger 2 positions -1–6) is shown. Finally, the desired binding specificity is confirmed for finger arrays that use a candidate finger that represents the main cluster.

To implement this intuition, the normalized Shannon entropy, as described in the Methods, was used as a measure of the diversity with which that particular amino acid sequence was observed. Amino acid sequences with multiple encodings and low normalized entropy (*E < 0.25*) were removed from further consideration. This computational filter significantly improved our ability to detect amino acid sequences that were enriched in selections (Figure 3). Finally, clusters of different types of entropy filtered ZFs selected to bind a common DNA target were determined by grouping proteins with high sequence similarity.

### Multiple cluster-designed ZFs offer desired specificity

**Figure 4.**
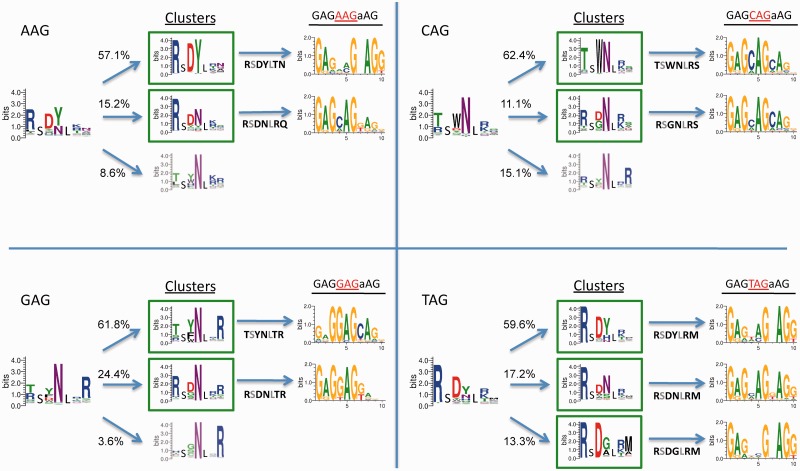
Selection of zinc fingers to bind all nAG targets. Zinc fingers were selected from the ‘RA’ library to bind all four nAG targets and processed as described in Figure 3 and in the text. In each case, the entropy filtered zinc fingers have been broken into multiple clusters, the largest three of which are shown. The percentage of the total sequences recovered that are included in each cluster is noted to the left. Boxed clusters were identified at low and high stringency. The specificity of candidates that represent these clusters was tested by B1H selection in the context of the three-finger protein using the C-terminal and N-terminal constant fingers (RSANLVR and RSDNL*RA*) used in the original finger selection. The sequence of the helix for each candidate zinc finger tested is shown under the arrow that points to the resulting specificity.

**Figure 5.**
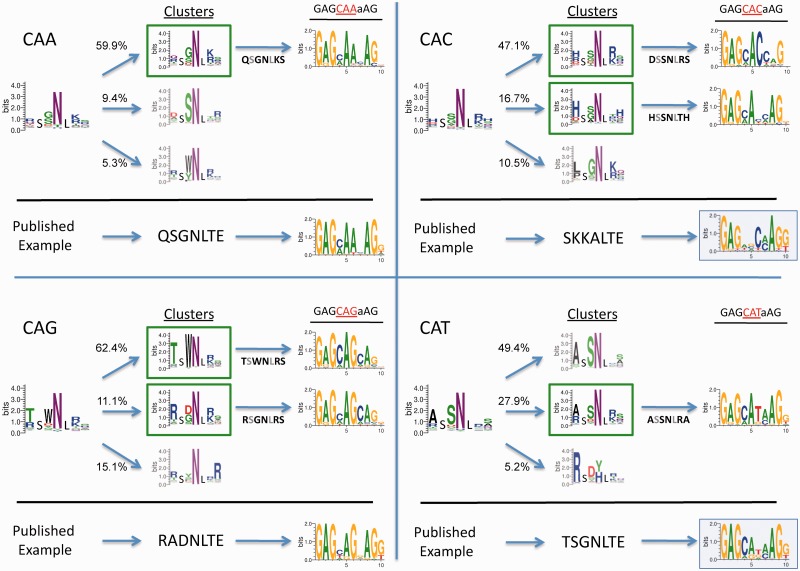
Selection of zinc fingers to bind all CAn targets. Zinc fingers were selected from the ‘RA’ library to bind all four CAn targets and processed as described in Figure 3 and in the text. In each case, the entropy filtered zinc fingers have been broken into multiple clusters, the largest three of which are shown. The percentage of the total sequences recovered that are included in each cluster is noted to the left. Boxed clusters were identified at low and high stringency. The specificity of candidates that represent these clusters were tested by B1H selection in the context of the three-finger protein using the C-terminal and N-terminal constant fingers (RSANLVR and RSDNL*RA*) used in the original finger selection. The sequence of the helix for each candidate zinc finger tested is shown under the arrow that points to the resulting specificity. Below each black line, the published helix suggested to bind each of these targets is also shown (67). Candidate proteins using these published helices were assembled and specificities determined.

ZFs were selected from the ‘RA’ library to bind all 5′ *n*AG 3′ and 5′ CA*n 3*′ targets by B1H selection (Figures 4 and 5, respectively). Entropy filtered ZFs were divided into clusters and the three clusters with the largest number of total sequence counts are shown. For each of the 5′ nAG 3′ and 5′ CAn 3′ targets, 4439–8006 protein sequences passed the entropy filter and were found in clusters of size at least 10. Clusters representing at least 5% of the total counts recovered at low stringency, and where a similar cluster was recovered at high stringency, were processed further and referred to as highly populated clusters. In the case of the 5′ CA*n* 3′ selections, 2 of 4 produced only one highly populated cluster (Figure 5, 5′ CAA 3′ and 5′ CAT 3′). Central ZFs were designed that resemble the highly populated clusters, and the DNA-binding specificity was determined by B1H selection in the context of the three-fingered protein they were originally selected with (Figure 5). The B1H method has been established as a simple approach to characterize specificities for all of the most commonly used DNA-binding domains, including ZFs; specificities produced by this method have high similarities to specificities determined by alternative methods (34,37,51,65,66). To provide a point of comparison, we also tested ZFs that have been suggested in the literature for the 5′ CA*n* 3′ targets (67). In most tested cases, the desired base preferences at the core 3-bp target are clearly reflected in the sequence logos corresponding to the determined DNA-binding specificities. In other cases, though the specificity offered by a candidate ZF might be weak, it is still evident that the finger would be able to bind the desired target. Interestingly, in many clusters, an aspartic acid is enriched at position 2 of the helix that has been shown to provide guanine or thymine preference at the base 3′ to the core 3 bp target. Though none of these ZFs were selected with a guanine or thymine at this position, either the guanine/thymine preference is noticeable or all specificity at the 3′ base is lost (Figure 4). Several ZFs selected in the RA libraries for the 5′ CA*n* 3′ clusters were also tested with an alternate N-terminal neighboring ZF, the N-terminal finger of *Zif268* (Supplementary Figure S4). Half of these fingers maintain their respective specificity with the alternative neighbor, whereas the other half either failed the selection or the specificity for the N-terminal finger is lost.

### Cluster-designed ZFs function as artificial TFs

**Figure 6.**
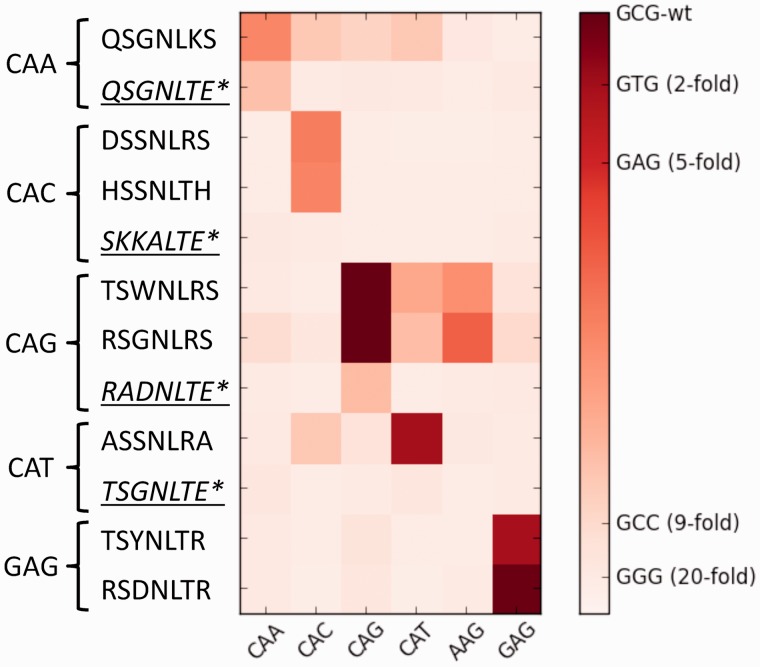
Artificial TF induction of GFP in yeast. We previously described a system to induce an artificial ZF-EV with β-estradiol (25). Activation of GFP can be induced with β-estradiol if a target complementary to the zinc finger used is placed in the promoter upstream of the GFP coding sequence. To provide a sense of relative specificity and affinity, the helices described in Figure 5, and the GAG-binding fingers described in Figure 4, were challenged with a set of similar targets, listed across the bottom of the chart. Each experiment included a positive control, the *Zif268* activity when paired with its consensus target. This allows for the GFP output to be normalized in each experiment to the positive control output. Here, the normalized GFP output for each zinc finger and binding site pair is shown as a heat plot. For reference, a key is provided that shows the normalized GFP output when *Zif268* is paired with binding sites of known affinity relative to the consensus (64). Asterisk: helices underlined and in italics were tested as suggested in the literature to bind the target noted on the left (67).

The three-fingered proteins described previously that harbor center fingers representing the helices tested to bind all 5′ CA*n* 3′ and 5′ GAG 3′ target were tested as artificial TFs. We have previously shown that induction of a ZF-EV by the addition of β-estradiol can activate a target gene site specifically with no significant background activity in yeast (25). We tested these cluster-designed ZF-EVs for their ability to activate a GFP reporter when the appropriate binding site is placed in the promoter. To provide a measure of specificity and relative affinity, we tested these same helices for their ability to induce GFP when paired with the set of targets 5′ CAA 3′, 5′ CAC 3′, 5′ CAG 3′, 5′ CAT 3′, 5′ AAG 3′ and 5′ GAG 3′ (Figure 6). For comparison, ZFs previously described to bind the 5′ CAn 3′ targets were also tested on this same set of targets (67). To provide an approximation of affinity, we also tested *Zif268* paired with a set of sequences where the N-terminal finger target has been modified. These targets offer known decreases in affinity relative to the *Zif268* consensus target (Figure 6, right) (64). All GFP outputs have been normalized to the experimental positive control, Zif268 paired with its consensus target, so a comparison can be made across experiments. All cluster-designed ZF-EVs activate their designed target significantly greater than any other target. By contrast, two helices suggested in the literature for their respective targets (SKKALTE-CAC and TSGNLTE-CAT) do not appear to have sufficient affinity to activate GFP when paired with any binding site; however, we note that these helices were selected in different contexts than the ones tested here. Complementary to the specificities offered in Figure 5, the 5′ CAG 3′ cluster-designed ZF’s also activate 5′ AAG 3′ strongly. Surprisingly these ZFs also activate the 5′ CAT 3′ target, indicating that two different strategies to bind a 3′ guanine will both tolerate tyrosine at this position.

## DISCUSSION

The method described here has the potential to greatly influence protein engineering, ZF engineering and as a result, genome engineering. Because our library building method is based on PCR assembly of a library cassette, it is easily modified for the construction of libraries for any protein of interest. As a result, our integrated pipeline for library building, characterizing diversity and determining enrichment based on coding entropy could be applied to any protein-engineering project of interest. Further, because deep sequencing is relatively easy and affordable, while all other steps require simple molecular biology techniques, our method should make it routine for a non-expert laboratory to build large diverse libraries of their protein of choice.

We have gone to great lengths to modify and document our library building procedure while confirming the reproducible diversity that it provides. By comparison, previous ZF library builds have typically sequenced a relatively small number of clones from the unselected library and confirmed a somewhat uniform distribution of nucleotides (17,50,52). Here, we characterize and confirm the diversity of our libraries by deep sequencing. The diversity of our libraries enables the selection of thousands of ZFs that bind a common target of interest, and that further cluster into distinct groups of sequences. Further, although it is intuitive to screen selected proteins for those encoded in a sufficiently diverse way, the success of this approach depends on the assumption that the starting library is itself diverse. A biased or incomplete library might offer a single coding sequence far in excess of the remaining library members. If this member is truly selected, our approach would discard this member because other ways of encoding this protein are not observed. Thus, the diversity of our libraries is necessary for the success of our overall approach.

To capture as many ZFs as possible able to bind each target, we reasoned that expressing the ZF library at a high level would allow for the recovery of ZFs that offer a wide range of affinities. To accomplish this goal we built our libraries with the previously described B1H expression vector with the strongest promoter (34). One risk with this type of approach is that a high background may result. However, by using the coding entropy we have shown that we can recover enriched proteins from our selections even when they are in the presence of a high background, whether that background is made of true or false positives, where true positives could represent competing clusters of ZFs.

By randomizing five amino acid positions, we can theoretically obtain 3.2 million possible ZFs, which is vastly larger than the 256 4-mer DNA subsites that any one of these fingers can bind. Thus, combinatorially, we expect that many individual ZFs bind each subsite. The ability to capture multiple distinct ZF clusters from a single selection, and in new selection contexts, could greatly enhance our ability to assemble engineered ZFs as modules. This is easily understood when one considers that with the exception of a few N-terminal ZF selections, most selections have used neighboring ZFs that would require guanine immediately 3′ to the 3 bp selection target (17,50,52,54,67). Not surprisingly, a strong enrichment for aspartic acid or glutamic acid at position 2 of the selected helix is often found as these residues have been shown to make a cross-strand contact with the cytosine that would complement the 3′ guanine to the selected target. This may be a fundamental limitation of the candidate ZFs often used for modular assembly and may help explain why the most successful assemblies have been those that offer repeats of overlapping GNN-binding fingers (13). 

The selections reported here use the ‘RA’ finger N-terminal to the selected finger, placing an adenine immediately 3′ to the 3-bp target. The ability to uncover multiple clusters of ZF proteins may be due to the use of the 3′ adenine, potential flexibility of the RA finger to accommodate multiple neighbors or simply the sequencing depth. In previously published reports the sequencing depth is not great enough to determine whether additional secondary clusters exist (17,50,52,54,67). However, we observe that our selections produce clusters that offer zinc fingers similar to those documented by these previous efforts as well as new types of fingers. In addition, the recovery of multiple clusters that use aromatic residues would not have been possible with the previous libraries that use a VNS coding scheme (52,54). Interestingly, the N-terminal OPEN selection of GAG, which as the N-terminal finger does not require a neighboring 3′ guanine, does not enrich for the aspartic acid or glutamic acid at position 2. Rather, a common finger enriched is TKHNLVR, which is similar to the first cluster selected here albeit without the aromatic at position 2 that the OPEN VNS code would not allow for (52). Finally, because previous fingers were selected with guanine-binding neighbors and functional assembly of these published GNN-binding fingers is well documented, it is possible that the fingers generated in this report will function under conditions that mimic their selection context as well (i.e. with either 3′ adenine specificity and/or neighbors similar to the ‘RA’ finger). Tests of these selected fingers with an alternative G-binding neighbor revealed that some fingers appear to provide a general solution to binding a triplet, whereas others seem more specialized. Therefore, providing multiple classes of zinc fingers per binding triplet has a clear advantage, as it provides multiple distinct alternatives to test. An expansion of the approach outlined in this manuscript to select ZFs with a suite of disparate neighbors may provide a guide to understanding generalized and specialized solutions and as a result, what types of ZFs assemble well together and what types do not. Regardless, more clusters able to bind common sequences provide more types of ZFs to choose from, each of which may provide a different set of neighbors with which it can function.

Though the work here is limited to a small number of targets in one selection context, further application of this approach may greatly improve our understanding of the ZF domain. As noted earlier in the text, this is one of the first examples of a fully randomized ZF selection where the base 3′ to the target was not a guanine. These new fingers were either not coded for or not found in previous libraries that used a guanine-binding neighbor and as a result extend our knowledge base of ZF protein–DNA interactions. Future selection of new fingers with alternative neighbors or at different positions within the parent protein may continue to provide details of the complex intra- and inter-finger influences that impact the protein–DNA interaction interface. We anticipate that an exhaustive portfolio of ZF clusters selected to bind every possible 3 bp target in multiple contexts and positions will be a great aid in improving our ability to predict and design the specificities of ZFs.

## SUPPLEMENTARY DATA

Supplementary Data are available at NAR Online.

## FUNDING

A.V.P. and M.S. were supported in part by NIH [R01 GM076275] and NSF [ABI 1062371]. E.F.R., B.L.O, and M.B.N. were supported by the Lewis-Sigler Fellow Program and endowed gift of Peter Lewis. Funding for open access charge: NIH [R01 GM076275] the Lewis-Sigler Fellow Program, endowed gift of Peter Lewis.

*Conflict of interest statement*. None declared.

## Supplementary Material

Supplementary Data
