# Supplementary material for: Deep sequencing of large library selections allows computational discovery of diverse sets of zinc fingers that bind common targets
Source: Nucleic Acids Res. 2013 Nov 7;42(3):1497–508. doi: 10.1093/nar/gkt1034 (PMC3919609; doi:10.1093/nar/gkt1034)
Supplement: Supplementary Data [file supp_42_3_1497__index.html]

Deep sequencing of large library selections allows computational discovery of diverse sets of zinc fingers that bind common targets — Deep sequencing of large library selections allows computational discovery of diverse sets of zinc fingers that bind common targets — Supplementary Data 

# Deep sequencing of large library selections allows computational discovery of diverse sets of zinc fingers that bind common targets

## Supplementary Data

files

**Files in this Data Supplement:**

- Supplementary Data - pdf file
- Supplementary Data - xlsx file
- Supplementary Data - docx file
