# Supplementary figures and images for: Deep sequencing of large library selections allows computational discovery of diverse sets of zinc fingers that bind common targets
Source: Nucleic Acids Res. 2013 Nov 7;42(3):1497–508. doi: 10.1093/nar/gkt1034 (PMC3919609; doi:10.1093/nar/gkt1034)

Figure S4

C - RTLNASR - TEST - ARLNDSR - N

C - RTLNASR - TEST - RTLEDSR - N

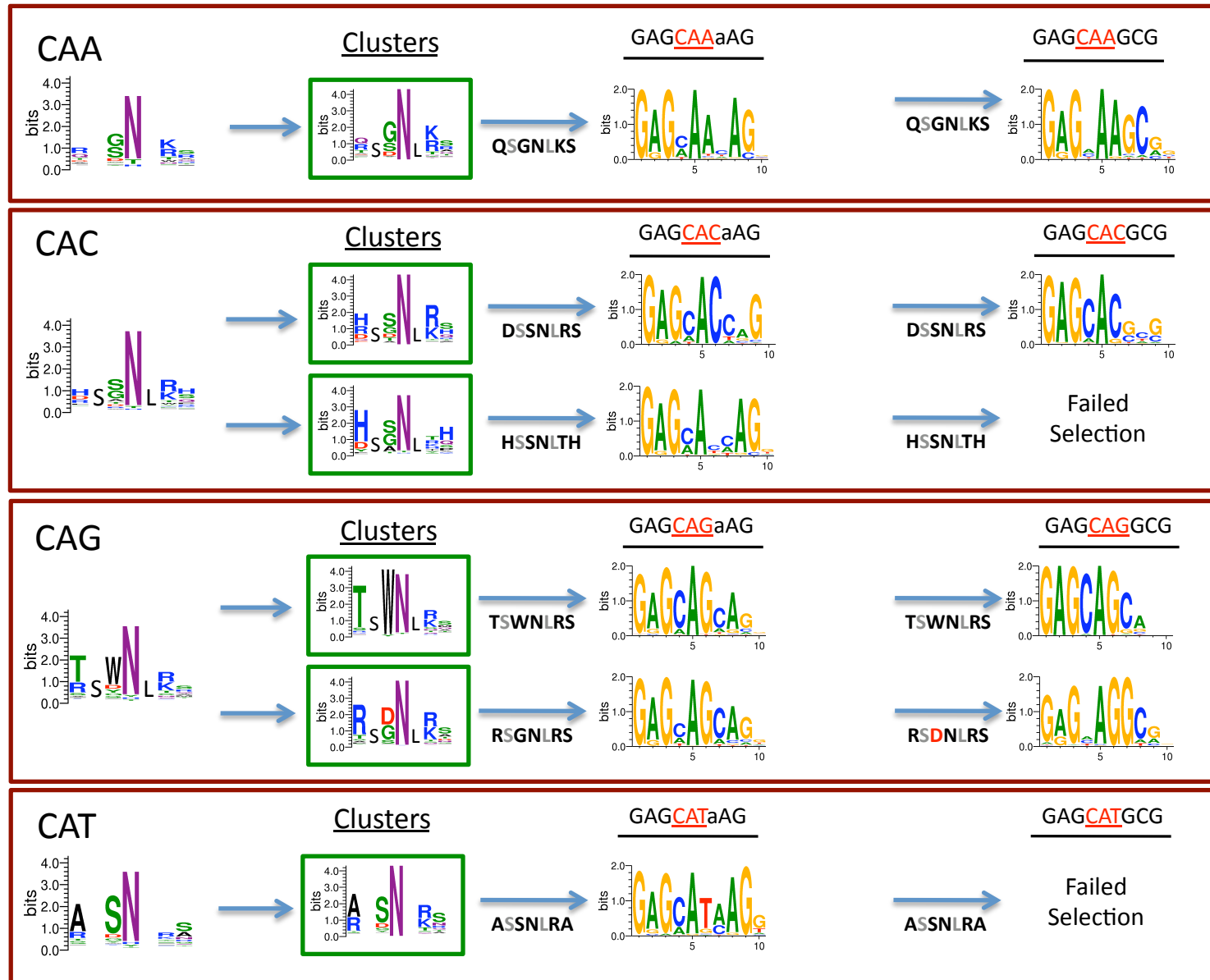

Supplement: Supplementary Data [file supp_gkt1034_nar-02486-z-2013-File013.pdf]
