# Supplementary material for: Deep sequencing of large library selections allows computational discovery of diverse sets of zinc fingers that bind common targets
Source: Nucleic Acids Res. 2013 Nov 7;42(3):1497–508. doi: 10.1093/nar/gkt1034 (PMC3919609; doi:10.1093/nar/gkt1034)
Supplement: Supplementary Data [file supp_gkt1034_nar-02486-z-2013-File009.docx]

Supplementary Information for:

Deep sequencing of large library selections allows computational discovery of diverse sets of zinc fingers that bind common targets

*Anton V. Persikov^1,*^, Elizabeth F. Rowland^1,*^, Benjamin L. Oakes^1^, Mona Singh^1,2,3†^, and Marcus B. Noyes^1,3†^*

^1^The Lewis-Sigler Institute for Integrative Genomics, Princeton University, Princeton, NJ 08544, USA.

^2^Department of Computer Science, Princeton University, Princeton, NJ 08544, USA

^3^Department of Molecular Biology, Princeton University, Princeton, NJ 08544, USA.

*These authors contributed equally to this manuscript.

**^†^** To whom correspondence may be addressed: [mona@cs.princeton.edu](mailto:mona@cs.princeton.edu), [mnoyes@princeton.edu](mailto:botstein@genomics.princeton.edu)

**Supplementary Table of Contents**

**A. Supplementary Figure Legends p2**

1. Assembly of large library cassettes
2. Library oligonucleotide annealing temperature impact on diversity
3. Library diversity is maintained throughout the library build

**B. Description of Supplementary Tables p3**

1. Table of oligonucleotides
2. Table of library build size
3. Oligonucleotide related codon bias
4. Number of colonies surviving the selections with the RA library

**C. Supplementary Method p3-8**

1. Library build protocol

2. B1H Selections

3. Illumina Preps

**D. Templates and Coding Sequences p8-12**

1. Three finger template

2. All library aa sequences

3. B1H expression vectors

**A. Supplementary Figure Legends**

**Figure S1**. Assembly of large library cassettes. Step 1. The library PCR fragment is amplified using a library oligonucleotide designed with the desired randomization scheme and taking the critical PCR parameters into account. In the example, the randomizations scheme (rainbow NNS) is designed to replace the desired codons to be sampled (red bases of template). The oligonucleotide also includes a 5’ SgrAI restriction site (green) used in the next step. Step 2. It is often advantageous to extend the length of a library PCR fragment. This is done by digesting the library cassette and the extension cassette with the appropriate restriction enzyme (SgrAI in the example). These digested fragments are gel purified and ligated together. The ligated, full-length library cassette is isolated from an agarose gel. Step 3. The full-length library cassette is amplified with external primers that will allow for cloning into the final expression vector. In the example we show primers that will code for the KpnI and XbaI restriction sites (blue) that will allow for proper cloning into our expression vectors. This expansion PCR is done in a 48-96 well format with 30 cycles to produce as much full-length library cassette as possible while minimizing the impact of potential jackpot events. The expanded cassette is finally digested with the external restriction enzymes (in the example, KpnI and XbaI) and cloned into the expression vector by large-scale ligation.

**Figure S2**. Library oligonucleotide annealing temperature impact on library diversity. The number of unique sequences recovered as a function of the number of total sequences processed is shown for libraries built with either 45 or 65^o^C annealing temperature. The theoretical line is a depiction of equation 1 in the main text representing the number of unique sequences one would expect from a truly random pool. The fraction of the theoretical maximum that is represented by the number of sequences processed is shown at the top. The fraction of expected diversity drops from over 95% to 81% when comparing 65 and 45^o^C annealing temperature. This is evident by comparing the distance between each data point and the theoretical line.

**Figure S3.** Library diversity is maintained throughout the library build. The number of unique sequences recovered as a function of the number of total sequences processed is shown for sequences recovered at 3 stages of the library build. Sequences were processed after the PCR fragments were ligated (Ligation), the PCR amplification of this ligated fragment (PCR Recovery) and after the final library build into the expression vector (Final Library). The theoretical line is a depiction of equation 1 in the main text representing the number of unique sequences one would expect from a truly random pool. The fraction of the theoretical maximum that is represented by the number of sequences processed is shown at the top. In each case the fraction of expected diversity is approximately 95% demonstrating maintenance of diversity throughout the process.

**Figure S4.** Candidate zinc fingers tested with an alternative neighbor.  Six candidate zinc fingers that represent the 5' CAn 3' binding clusters were tested when expressed with an alternative N-terminal neighbor, the N-terminal finger of *Zif268* (N-RSDELTR) which specifies 5' GCG 3'. The clusters from which these fingers were designed and the specificities determined with the original anchor are provided (see Figure 5). To the right, the specificities of these fingers determined with the *Zif268* anchor is provided. Any B1H selection that failed to produce at least 5-fold the number of colonies in comparison to a negative control was considered a failed selection and not processed further. The second CAG-binding cluster allows a D or G at position 2 of the helix.  Since a D at this position has been shown to accommodate G/T specificity at the overlap position, this amino acid was used in the *Zif268* neighbor candidate.

**B. Description of Supplementary Tables**

1. Table of oligonucleotides used in this paper
2. Table of library sizes

- Library build size for all 12 libraries described in this paper

1. Oligonucleotide related codon bias

- Comparison of F2 and F3 5aa library builds for base bias at each position, based on an N or S coding scheme. Demonstrates that some bias follow trends in positions common to libraries built with common oligonucleotides. This implies that at least some of the small bias found in the libraries may be inherent to the oligonucleotide synthesis, not the library build.

1. Zinc finger selection success from the RA library

- Table of the number of colonies surviving selections to bind each target

5. GFP Fluorescence measurement for yeast, artificial transcription factor assay in Figure 6.

**C. Supplementary Method**

**Library build protocol**

**Designing the oligonucleotide**

There are 3 critical regions of the oligonucleotides used for library construction:

The annealing region, the library, and the extension (see Figure S1 for reference).

**The annealing region** is the region 3-prime to the random region of the oligo and is designed to have 15-20^o^C of annealing temperature for the template DNA that is ***above*** the temperature that will be used in the PCR reaction. We typically design the annealing region of the oligo to have 65-70^o^C of salt-adjusted annealing temperature, while we run the PCR reactions at 50^o^C during the annealing step. This should minimize any impact that any given library member might have on the efficiency of annealing in the PCR reaction which could result in bias.

**The library**, random region of the oligo is designed to introduce the desired randomization scheme at the exact residues of interest. In our case, we have used an NNS coding scheme for each codon to be randomized because this scheme will code for every possible amino acid and one stop codon. We also order these oligos from IDT using their “hand-mixed” specification as opposed to their machine-mixed option. Hand-mixing the random bases takes the coupling constants into account when the oligos are synthesized and, in our experience, has seemed to produce a more true distribution of bases at the desired positions.

**The extension** is the region of the oligo 5 prime to the library. This region is important for the capture of the library. To install the library into an expression vector, or to attach it to a larger DNA fragment (see below in library PCR assembly) this region is critical. There are alternative approaches to capturing the library at this phase but the approach used here has been to capture the library PCR by digestion and ligation with an extended piece of DNA. Since oligos are assembled 3’ to 5’, this ensures that every captured piece of DNA from the library PCR reaction is full-length, containing the restriction site at the 5’ end of the oligo.

**Library Build Procedure**

There are 5 steps to build a library: 1. library PCR round 1, 2. digestion/ligation Capture, 3. library PCR round 2, 4. digestion for ligation into expression construct and ligation, and 5. transformation and expansion. Steps 4 and 5 follow what is detailed in Figure 1. These last steps are no different from what one would do by traditional cassette mutagenesis, just with a great deal more cassette to work with.

**Library PCR round 1**. Library oligos are designed as detailed above. 48-96 PCR reactions are set up using these oligos and the template that will install the appropriate anchor fingers. Our template is provided below in the supplementary information, it is the *Zif268* backbone with silent alterations that allow the installation of various restriction sites. 48-96 reactions are used to minimize any influence that a PCR “jackpot” event might have on the total library. For example, if one PCR reaction turned out to be massively biased, when pooled with the remainder of the material, that bias still represents only 1% of the total library.

Each PCR reaction uses Expand High Fidelity Plus (Roche, 04 743 733 001) and is set up as listed in the supplementary protocols. The critical parameters are setting up the reaction to be run 20^o^C below the oligo annealing temperature and limiting this step to 15-20 cycles. PCR reactions are pooled and recovered by gel electrophoresis and gel extraction.

**Digestion/Ligation Capture**. The recovered DNA is digested with the appropriate enzyme to assemble with the desired extension fragment. In our case, SgrAI. The finger 2 library oligo will need to have amplified the randomized finger 2 as well as the desired anchor finger at position 1. Therefore, each of our libraries required the construction of a template to amplify from that codes for a different anchor finger in the finger 1 positions. These anchors are listed in Supplementary Table 3. To assemble, the Finger 3 anchor, the RSANLVR finger, is amplified as with the complimentary primer (this does not need to be done in a 96 well format or limited to 15 cycles). The F2 Library/finger 1 fragment and F3 PCR fragments are then digested with SgrAI (F3 libraries also used SgrAI with the primer in the reverse orientation). This was done according to manufacturers guidelines, 2 hours at 37^o^C. Digests were recovered by gel electrophoresis and gel extraction. Finally, T4 DNA ligations were set up per manufacturers guidlines with a 1:1 molar equivalent of each fragment, in a small enough volume to be run on 1 or 2 lanes of an agarose gel. The resulting, ligated band was 440 base pairs. This band was recovered from the gel.

**Library PCR round 2**

The ligated library material is in the proper format but is not enough for a large-scale library build. To expand the material while minimizing any potential bias, the ligated material is expanded by a 2^nd^ round of PCR. This PCR reaction uses the external primers that are far removed from the random region of the DNA fragment. In our case, these were the OMG5 and OK60 primers (Supplementary Table 1). Therefore, the sequence of any given library member should have no influence on the efficiency of the PCR reaction and this should result in a true expansion of the DNA without bias. PCR reactions were set up again in a 96-well format to minimize the influence of a potential “jackpot” event. Details of the reaction are noted in the supplementary protocol. The critical parameters were to run 30 cycles of the reaction using 50^o^C annealing temperature. PCR reactions were pooled and recovered by gel electrophoresis and gel extraction.

**Digestion for expression construct ligation.** The recovered PCR material from the 2^nd^, expansion round of PCR is digested with KpnI and XbaI or BamHI according to the manufacturer’s guidelines. These restriction sites were chosen simply because they accommodate ligation into our parent expression vectors. These sites could certainly be modified to accommodate any application desired by a researcher. The digested material was recovered by gel electrophoresis.

T4 DNA Ligase was used for large-scale library ligation. Here, 2ug of the Kpn1-XbaI digested, expression vector is used per 20ul ligation. For our libraries 5-10 ligations were performed using a 5:1 molar ratio of insert to vector. Therefore 5-10, 20ul ligations as detailed here lead to a total, 20ug, 200ul ligation. Ligations were held at 16^o^C for 12-14 hours, followed by a 20-minute heat-kill at 65^o^C. Finally, ligations were ethanol precipitated and resuspended in 20ul of Tris-buffer. Therefore, if one assumes zero loss, 1ul = 1ug ligation.

**Transformation and expansion**. A test transformation of 1ul of the recovered ligation above was always tested to determine how many transformants would be recovered from a single transformation. In most cases, the result was 5x10^8^ – 1x10^9^. Therefore, 10 transformations were set up for each library build, each using a single ul of the ligation. Transformations used 75ul of electrocompetent E.coli (as prepped below) and 1ul of the ligation. Upon electroporation, the transformations were recovered in 1L SOC (SOB+0.5% glucose). For controls, an empty ligation using 1ug of backbone and no insert served as a control for the ligation fold over the background. This transformation was recovered in 100ml SOC representing 1/10^th^ the library build and therefore 1/10^th^ the volume. For expansion control, a transformation without DNA was also performed and recovered in 100ml SOC. After electroporation, cells were recovered for 45-minutes at 37^o^C with shaking. 200ul were recovered from each control and the library build. These 200ul were plated on antibiotic selective plates in 10-fold serial dilutions to determine library size (Supplementary Table 1). AT this point carbenicillin was added to each culture to “select” for the presence of the ligated plasmid. Cells were allowed to expand to an OD600 of 0.5-0.75 when using the “expansion control” as the blank (approximately 5-6 hours). This blank removes cell debris from the calculation and only measures cells that have been expanding with carbenicillin. DNA was harvested from the Liter of library expansion and sequenced to confirm diversity.

**Calculating Library Build**

The following day, the number of cells that took up plasmid are calculated from the serial-dilution plates. Since this number is taken from a time point before the library has expanded, this number represents library build size. In no case did we find cells that survived on carbenicillin in the expansion control since no DNA was added. When comparing the library ligation to the no insert control, we typically find roughly 100-fold increase in the number of transformants per unit plated for the library insert relative to the control. However, its worth noting that this fold over the background is largely dependant on the quality of the digestion of the vector used by the researcher. It is therefore up to the researcher to determine what is a reasonable amount of background to be tolerated in their particular application. We were satisfied with the 100-fold over the background demonstrated here because it approximates that roughly 1% of our total library build is from background and 99% is from ligated library material.

**Electrocompetent Cell Preparation**

For library builds and selections we use an *E. coli* cell line previously constructed for the bacterial one-hybrid system and available through addgene([1](#_ENREF_1)). This line is required for selections but is not required for library building. We use this line for libraries because it consistently demonstrates high transformation efficiency and hasn’t impacted the diversity recovered in our libraries in any noticeable way. However, libraries could be built by this approach using any cell line desired by the end user. Still it must be noted that the library build size is influenced by transformation efficiency so the electrocompetency of the strain utilized is extremely important. Our cell line has been made electrocompetent as follows.

1L of of sterile 2xYT and 10μg/ml tetracycline is inoculated with one starter culture. A starter culture is a 1ml glycerol stock of the cells grown to OD600 of 0.6, concentrated to 30 aliquots of 1ml and flash frozen. The 1L culture is inoculated with one starter culture and grown with shaking at 37^o^C. When the culture reaches an OD600 of approximately 0.5-0.6, the culture is quickly cooled by swirling in an ice bath until the culture is a uniform 4^o^C (10-20 minutes). The culture is split into 4 pre-chilled 250ml disposable Corning conical bottom tubes. Next, the culture is pelleted by centrifugation in a pre-chilled rotor. We use the J5.3 rotor in Beckman Coulter Avanti J-E centrifuge. The 4 conical bottom tubes are spun down at 5000 rpm for 12 minutes. Decant supernatant and re-suspend pellet in 20mL, pre-chilled sterile water, combine the 4 pellets into 2 flasks. Fill flasks with sterile water up to 225mL. Spin down at 4000 rpm for 10 minutes. Decant the supernatant and repeat the sterile water wash and spin down at 4000 rpm again. Decant supernatant, resuspend and wash with 150mL 10% (w/v) glycerol for each conical, spin down at 4000 rpm for 10 minutes. Decant supernatant and re-suspend the pellets in 20mL 10% (w/v) glycerol. Combine the 2 pellets into one flask, fill with 10% (w/v) glycerol up to 125mL. Spin down at 4000 rpm, for 13 min. Completely decant the supernatant. Resuspend pellet in 2.75mL 10%(w/v) glycerol. Aliquot 88μL of cell suspension into labeled micro-centrifuge tubes and flash freeze on dry ice. Once cell suspension is frozen, store at -80°C. 75ul of one aliquot of cells is used per transformation.

**Zinc finger selection**

Zinc finger selections were performed as previously described([2-4](#_ENREF_2)). Briefly, the appropriate zinc finger library was transformed along with the binding site, reporter plasmid (pH3U3) to be assayed (see Supplementary Table 1 for list of oligonucleotides used). The binding site reporters are built by placing the binding site of interest 10bp upstream of the -35box that drives HIS3 expression. These sites are cloned between the Not1 and EcoRI sites in the plasmid. After transformation with both plasmids, the cells were expanded, washed and counted by serial dilution. After storing the cells overnight at 4^o^C, the serial dilutions were counted and roughly 1x10^8^ cells plated on selective media. These plates contained low (2.5mM) and high (10mM or 25mM) 3-aminotriazole (3-AT), which is a competitive inhibitor of HIS3. The 3-AT is used to challenge activation of HIS3 and require a stringent protein-DNA interaction. Cell were grown on the selection plates for 36-48 hours at 37^o^C. Surviving cells were counted and pooled, prepped for illumina sequencing.

**Binding site selection**

Zinc finger binding site selections were performed as previously described([3](#_ENREF_3),[5](#_ENREF_5)). Briefly, an individual zinc finger expression vector was transformed along with a reporter plasmid library (pH3U3) that contains a 28bp region or randomized sequences upstream of the -35 box. This library has been previously described([1](#_ENREF_1)). Transformants are treated as above and grown on selective plates for 36-48 hours. Surviving cells were counted, pooled, and prepped for illumina sequencing. For a detailed description of the bacterial one-hybrid procedure and its application see methods reference 3.

**Illumina Prep**

Either zinc finger selections or binding site selections were prepped for illumina sequencing as follows. After counting, cells were pooled from selection plates and the plasmid DNA was recovered. The variable region of either the zinc fingers or the binding sites were amplified by PCR using the illumina primers listed in Table S4. The PCR reactions used Expand High Fidelity Plus (Roche, 04 743 733 001) and were typically set up in 96-well plates. A 50µL illumina PCR reaction for each DNA pool was set up:

10µL 5x Expand HiFi Reaction Buffer

1 µL 200 µM PCR Grade Nucleotide Mix

1 µL of 25 µM upstream, Barcode primer

1 µL of 25 µM downstream primer

0.5 µL of Expand HiFi Plus Enzyme Blend (5U/µL)

2 µL of DNA pool template

34.5 µL sterile H20

50 µL

The 96-well plate was heat-sealed and PCR reaction performed:

PCR conditions are set at:

94C – 2 minutes

12 cycles of the following

94C – 30 seconds

50C – 30 seconds

72C – 30 Seconds

72C – 10 minutes final polishing step

Hold at 4C

Once the PCR reactions finished, they were run out on a 1.5% (w/v) agarose gel and recovered using a gel extraction kit (Qiagen, 28606). The DNA was eluted from the Qiagen column in 8 µL of elution buffer. Each product was measured (Thermo scientific, Nanodrop 2000c) and diluted to 10nM. The products were combined and sequenced.

**D. Templates and Coding sequences**

**Backbone coding sequence for all zinc fingers and libraries:**

*GGTACC*GAGCGCCCATATGCTTGCCCTGTCGAGTCCTGCGATCGCCGCTTTTCTNNSAGTNNSNN

*KpnI*

SCTTNNSNNSCATATCCGCATCCAT*ACCGGT*CAGAAGCCCTTCCAGTGTCGAATCTGCATGCGTA

*AgeI*

ACTTCAGTNNSAGTNNSNNSCTTNNSNNSCACATCCGCACCCA*CACCGGCG*AGAAGCCTTTTGCC

*SgrAI*

TGTGACATTTGTGGGAGGAAGTTTGCCNNSAGTNNSNNSCTGNNSNNSCATACCAAAATCCATCT

CCGC*GGATCC*TAAG*TCTAGA*

*BamHI XbaI*

*Restriction sites used for cloning and library builds are color coded in red. The recognition region of the helix for each finger is color coded green. NNS codons are used here to show the randomization scheme for 5 amino acids per zinc finger where the Ser and Leu at positions 1 and 4 of the helices are fixed.*

**Amino acid sequence for each constructed ZF library**

*The eight, 5 amino acid libraries are listed below. The anchor finger the differentiates each library is color coded blue. X=NNS randomized codon. Target sequences are listed in parenthesis. Lib # corresponds to Figure 2 of the main text.*

**RA-library (5’-GAG NNN (a/c)AG-3’)**

GTERPYACPVESCDRRFS**RSDNLRA**HIRIHTGQKPFQCRICMRNFS***XSXXLXX***HIRTHTGEKPFACDICGRKFA**RSANLVR**HTKIHLRGS

**H4-library, Lib 1 (5’-GAG NNN ATA-3’)**

GTERPYACPVESCDRRFS**SSQGLAQ**HIRIHTGQKPFQCRICMRNFS***XSXXLXX***HIRTHTGEKPFACDICGRKFA**RSANLVR**HTKIHLRGS

**D5-library, Lib 2 (5’-GAG NNN CCA-3’)**

GTERPYACPVESCDRRFS**SSNSLYY**HIRIHTGQKPFQCRICMRNFS***XSXXLXX***HIRTHTGEKPFACDICGRKFA**RSANLVR**HTKIHLRGS

**D8-library, Lib 3 (5’-GAG NNN taC-3’)**

GTERPYACPVESCDRRFS**DVSNLKK**HIRIHTGQKPFQCRICMRNFS***XSXXLXX***HIRTHTGEKPFACDICGRKFA**RSANLVR**HTKIHLRGS

**Zif-library, Lib 5 (5’-GAG NNN GCG-3’)**

GTERPYACPVESCDRRFS**RSDELTR**HIRIHTGQKPFQCRICMRNFS***XSXXLXX***HIRTHTGEKPFACDICGRKFA**RSANLVR**HTKIHLRGS

**RAc-library, Lib 4 (5’-NNN GAG (a/c)AG-3’)**

GTERPYACPVESCDRRFS**RSDNLRA**HIRIHTGQKPFQCRICMRNFS**RSANLVR**HIRTHTGEKPFACDICGRKFA***XSXXLXX***HTKIHLRGS

**D8c-library, Lib 6 (5’-NNN GAG taC-3’)**

GTERPYACPVESCDRRFS**DVSNLKK**HIRIHTGQKPFQCRICMRNFS**RSANLVR**HIRTHTGEKPFACDICGRKFA***XSXXLXX***HTKIHLRGS

**Zifc-library, Lib 7 (5’-NNN GAG GCG-3’)**

GTERPYACPVESCDRRFS**RSDELTR**HIRIHTGQKPFQCRICMRNFS**RSANLVR**HIRTHTGEKPFACDICGRKFA***XSXXLXX***HTKIHLRGS

*The four, 6 amino acid libraries are listed below. The anchor fingers and fixed contacts are bold and black. Green X = NNS randomized codon. Target sequences are listed in parenthesis. Lib # corresponds to Figure 2 of the main text.*

**F1 library (5’-aaG GGG NNN-3’)**

GTERPYACPVESCDRRFS***XXXXLXX***HIRIHTGQKPFQCRICMRNFS**RSAHLVR**HIRTHTGEKPFACDICGRKFA**QRRYLRA**HTKIHLRGS

**F2 library (5’-GAG NNN (a/c)AG-3’)**

GTERPYACPVESCDRRFS**RSDNLRA**HIRIHTGQKPFQCRICMRNFS***XXXXLXX***HIRTHTGEKPFACDICGRKFA**RSANLVR**HTKIHLRGS

**F3 library (5’-NNN (a/c)AG GCG-3’)**

GTERPYACPVESCDRRFS**RSDELTR**HIRIHTGQKPFQCRICMRNFS**RSDNLRA**HIRTHTGEKPFACDICGRKFA***XXXXLXX***HTKIHLRGS

**F2-3 library (5’-GNN NNG GCG-3’)**

GTERPYACPVESCDRRFS**RSDELTR**HIRIHTGQKPFQCRICMRNFS**RSD*X*L*XX***HIRTHTGEKPFACDICGRKFA***X*S*XX*LTR**HTKIHLRGS

Expression vectors

Strong Promoter:

pB1H2wL – Zif Recode

GAATTCAAAAAAATATTGACAACATAAAAAACTTTGTGTTATACTTGTAACGCTACATGGAGATTAACTCAATCTAGCTAGAGAGGCTTTACACTTTATGCTTCCGGCTCGTATAATGTGTGGAATTGTGAGCGGATAACAATTTCACACAGGAAACAGCTATGACCATGATTACGGATTCACTGGAACTCTAAACCAAAGAGAGGACACCATGGCACGCGTAACTGTTCAGGACGCTGTAGAGAAAATTGGTAACCGTTTTGACCTGGTACTGGTCGCCGCGCGTCGCGCTCGTCAGATGCAGGTAGGCGGAAAGGACCCGCTCGTACCGGAAGAAAACGATAAAACCACTGTAATCGCGCTGCGCGAAATCGAAGAAGGTCTGATCAACAACCAGATCCTCGACGTTCGCGAACGCCAGGAACAGCAAGAGCAGGAAGCCGCTGAATTACAAGCCGTTACCGCTATTGCTGAAGGTCGTGCGGCCGCGGACTACAAGGATGACGACGACAAGTTCCGGACCGGTTCCAAGACACCCCCCCATGGTACCGAGCGCCCATATGCTTGCCCTGTCGAGTCCTGCGATCGCCGCTTTTCTCGCTCGGATGAGCTTACCCGCCATATCCGCATCCATACCGGTCAGAAGCCCTTCCAGTGTCGAATCTGCATGCGTAACTTCAGTCGTAGTGACCACCTTACCACCCACATCCGCACCCACACCGGCGAGAAGCCTTTTGCCTGTGACATTTGTGGGAGGAAGTTTGCCAGGAGTGATGAACGCAAGAGGCATACCAAAATCCATCTCCGCGGATCCTAAGTCTAGAGACTAGAAAAAGGCCGACAAGTCCCGCTCCGCTGAAGATCCTGGCGTAATAGCGAAGAGGCCCGCACCGATCGCCCTTCCCAACAGTTGCGCAGCCTGAATGGCGAATGGGACGCGCCCTGTAGCGGCGCATTAAGCGCGGCGGGTGTGGTGGTTACGCGCAGCGTGACCGCTACACTTGCCAGCGCCCTAGCGCCCGCTCCTTTCGCTTTCTTCCCTTCCTTTCTCGCCACGTTCGCCGGCTTTCCCCGTCAAGCTCTAAATCGGGGGCTCCCTTTAGGGTTCCGATTTAGTGCTTTACGGCACCTCGACCCCAAAAAACTTGATTAGGGTGATGGTTCACGTAGTGGGCCATCGCCCTGATAGACGGTTTTTCGCCCTTTGACGTTGGAGTCCACGTTCTTTAATAGTGGACTCTTGTTCCAAACTGGAACAACACTCAACCCTATCTCGGTCTATTCTTTTGATTTATAAGGGATTTTGCCGATTTCGGCCTATTGGTTAAAAAATGAGCTGATTTAACAAAAATTTAACGCGAATTTTAACAAAATATTAACGCTTACAATTTAGGTGGCACTTTTCGGGGAAATGTGCGCGGAACCCCTATTTGTTTATTTTTCTAAATACATTCAAATATGTATCCGCTCATGAGACAATAACCCTGATAAATGCTTCAATAATATTGAAAAAGGAAGAGTATGAGTATTCAACATTTCCGTGTCGCCCTTATTCCCTTTTTTGCGGCATTTTGCCTTCCTGTTTTTGCTCACCCAGAAACGCTGGTGAAAGTAAAAGATGCTGAAGATCAGTTGGGTGCACGAGTGGGTTACATCGAACTGGATCTCAACAGCGGTAAGATCCTTGAGAGTTTTCGCCCCGAAGAACGTTTTCCAATGATGAGCACTTTTAAAGTTCTGCTATGTGGCGCGGTATTATCCCGTATTGACGCCGGGCAAGAGCAACTCGGTCGCCGCATACACTATTCTCAGAATGACTTGGTTGAGTACTCACCAGTCACAGAAAAGCATCTTACGGATGGCATGACAGTAAGAGAATTATGCAGTGCTGCCATAACCATGAGTGATAACACTGCGGCCAACTTACTTCTGACAACGATCGGAGGACCGAAGGAGCTAACCGCTTTTTTGCACAACATGGGGGATCATGTAACTCGCCTTGATCGTTGGGAACCGGAGCTGAATGAAGCCATACCAAACGACGAGCGTGACACCACGATGCCTGTAGCAATGGCAACAACGTTGCGCAAACTATTAACTGGCGAACTACTTACTCTAGCTTCCCGGCAACAATTAATAGACTGGATGGAGGCGGATAAAGTTGCAGGACCACTTCTGCGCTCGGCCCTTCCGGCTGGCTGGTTTATTGCTGATAAATCTGGAGCCGGTGAGCGTGGGTCTCGCGGTATCATTGCAGCACTGGGGCCAGATGGTAAGCCCTCCCGTATCGTAGTTATCTACACGACGGGGAGTCAGGCAACTATGGATGAACGAAATAGACAGATCGCTGAGATAGGTGCCTCACTGATTAAGCATTGGTAACTGTCAGACCAAGTTTACTCATATATACTTTAGATTGATTTAAAACTTCATTTTTAATTTAAAAGGATCTAGGTGAAGATCCTTTTTGATAATCTCATGACCAAAATCCCTTAACGTGAGTTTTCGTTCCACTGAGCGTCAGACCCCGTAGAAAAGATCAAAGGATCTTCTTGAGATCCTTTTTTTCTGCGCGTAATCTGCTGCTTGCAAACAAAAAAACCACCGCTACCAGCGGTGGTTTGTTTGCCGGATCAAGAGCTACCAACTCTTTTTCCGAAGGTAACTGGCTTCAGCAGAGCGCAGATACCAAATACTGTCCTTCTAGTGTAGCCGTAGTTAGGCCACCACTTCAAGAACTCTGTAGCACCGCCTACATACCTCGCTCTGCTAATCCTGTTACCAGTGGCTGCTGCCAGTGGCGATAAGTCGTGTCTTACCGGGTTGGACTCAAGACGATAGTTACCGGATAAGGCGCAGCGGTCGGGCTGAACGGGGGGTTCGTGCACACAGCCCAGCTTGGAGCGAACGACCTACACCGAACTGAGATACCTACAGCGTGAGCTATGAGAAAGCGCCACGCTTCCCGAAGGGAGAAAGGCGGACAGGTATCCGGTAAGCGGCAGGGTCGGAACAGGAGAGCGCACGAGGGAGCTTCCAGGGGGAAACGCCTGGTATCTTTATAGTCCTGTCGGGTTTCGCCACCTCTGACTTGAGCGTCGATTTTTGTGATGCTCGTCAGGGGGGCGGAGCCTATGGAAAAACGCCAGCAACGCGGCCTTTTTACGGTTCCTGGCCTTTTGCTGGCCTTTTGCTCACATGTTCTTTCCTGCGTTATCCCCTGATTCTGTGGATAACCGTATTACCGCCTTTGAGTGAGCTGATACCGCTCGCCGCAGCCGAACGACCGAGCGCAGCGAGTCAGTGAGCGAGGAAGCGGAAGAGCGCCTGATGCGGTATTTTCTCCTTACGCATCTGTGCGGTATTTCACACCGCATATGGTGCACTCTCAGTACAATCTGCTCTGATGCCGCATAGTTAAGCCAGTATACACTCCGCTATCGCTACGTGACTGGGTCATGGCTGCGCCCCGACACCCGCCAACACCCGCTGACGCGCCCTGACGGGCTTGTCTGCTCCCGGCATCCGCTTACAGACAAGCTGTGACCGTCTCCGGGAGCTGCATGTGTCAGAGGTTTTCACCGTCATCACCGAAACGCGCGAGGCAGCTGCGGTAAAGCTCATCAGCGTGGTCGTGAAGCGATTCACAGATGTCTGCCTGTTCATCCGCGTCCAGCTCGTTGAGTTTCTCCAGAAGCGTTAATGTCTGGCTTCTGATAAAGCGGGCCATGTTAAGGGCGGTTTTTTCCTGTTTGGTCACTGATGCCTCCGTGTAAGGGGGATTTCTGTTCATGGGGGTAATGATACCGATGAAACGAGAGAGGATGCTCACGATACGGGTTACTGATGATGAACATGCCCGGTTACTGGAACGG

Medium Promoter:

pB1H2w5 – Zif Recode

GAATTCCGGGCTTTACACTTTATGCTTCCGGCTCGTATAATGTGTCGACTTGTGAGCGGATAACAATTTCACACAGGAAACAGCTATGCCTCAACAGCAGCAAATGCAACCTCCCAATTCAAGTGCGGACAACAACCCTTTGCAACAGCAATCATCACAAAATACCGTACCAAACGTCCTCAACCAAATTAACCAAATCTTTTCTCCAGAGGAGCAACGCAGCTTATTACAAGAAGCCATCGAAACCTGCAAGAATTTTGAAAAAACACAATTGTAAGTCTCGGCTCCAAGAAGGAGATATACCCATGGCACGCGTAACTGTTCAGGACGCTGTAGAGAAAATTGGTAACCGTTTTGACCTGGTACTGGTCGCCGCGCGTCGCGCTCGTCAGATGCAGGTAGGCGGAAAGGACCCGCTCGTACCGGAAGAAAACGATAAAACCACTGTAATCGCGCTGCGCGAAATCGAAGAAGGTCTGATCAACAACCAGATCCTCGACGTTCGCGAACGCCAGGAACAGCAAGAGCAGGAAGCCGCTGAATTACAAGCCGTTACCGCTATTGCTGAAGGTCGTGCGGCCGCGGACTACAAGGATGACGACGACAAGTTCCGGACCGGTTCCAAGACACCCCCCCATGGTACCGAGCGCCCATATGCTTGCCCTGTCGAGTCCTGCGATCGCCGCTTTTCTCGCTCGGATGAGCTTACCCGCCATATCCGCATCCATACCGGTCAGAAGCCCTTCCAGTGTCGAATCTGCATGCGTAACTTCAGTCGTAGTGACCACCTTACCACCCACATCCGCACCCACACCGGCGAGAAGCCTTTTGCCTGTGACATTTGTGGGAGGAAGTTTGCCAGGAGTGATGAACGCAAGAGGCATACCAAAATCCATCTCCGCGGATCCTAAGTCTAGAGACTAGAAAAAGGCCGACAAGTCCCGCTCCGCTGAAGATCCTGGCGTAATAGCGAAGAGGCCCGCACCGATCGCCCTTCCCAACAGTTGCGCAGCCTGAATGGCGAATGGGACGCGCCCTGTAGCGGCGCATTAAGCGCGGCGGGTGTGGTGGTTACGCGCAGCGTGACCGCTACACTTGCCAGCGCCCTAGCGCCCGCTCCTTTCGCTTTCTTCCCTTCCTTTCTCGCCACGTTCGCCGGCTTTCCCCGTCAAGCTCTAAATCGGGGGCTCCCTTTAGGGTTCCGATTTAGTGCTTTACGGCACCTCGACCCCAAAAAACTTGATTAGGGTGATGGTTCACGTAGTGGGCCATCGCCCTGATAGACGGTTTTTCGCCCTTTGACGTTGGAGTCCACGTTCTTTAATAGTGGACTCTTGTTCCAAACTGGAACAACACTCAACCCTATCTCGGTCTATTCTTTTGATTTATAAGGGATTTTGCCGATTTCGGCCTATTGGTTAAAAAATGAGCTGATTTAACAAAAATTTAACGCGAATTTTAACAAAATATTAACGCTTACAATTTAGGTGGCACTTTTCGGGGAAATGTGCGCGGAACCCCTATTTGTTTATTTTTCTAAATACATTCAAATATGTATCCGCTCATGAGACAATAACCCTGATAAATGCTTCAATAATATTGAAAAAGGAAGAGTATGAGTATTCAACATTTCCGTGTCGCCCTTATTCCCTTTTTTGCGGCATTTTGCCTTCCTGTTTTTGCTCACCCAGAAACGCTGGTGAAAGTAAAAGATGCTGAAGATCAGTTGGGTGCACGAGTGGGTTACATCGAACTGGATCTCAACAGCGGTAAGATCCTTGAGAGTTTTCGCCCCGAAGAACGTTTTCCAATGATGAGCACTTTTAAAGTTCTGCTATGTGGCGCGGTATTATCCCGTATTGACGCCGGGCAAGAGCAACTCGGTCGCCGCATACACTATTCTCAGAATGACTTGGTTGAGTACTCACCAGTCACAGAAAAGCATCTTACGGATGGCATGACAGTAAGAGAATTATGCAGTGCTGCCATAACCATGAGTGATAACACTGCGGCCAACTTACTTCTGACAACGATCGGAGGACCGAAGGAGCTAACCGCTTTTTTGCACAACATGGGGGATCATGTAACTCGCCTTGATCGTTGGGAACCGGAGCTGAATGAAGCCATACCAAACGACGAGCGTGACACCACGATGCCTGTAGCAATGGCAACAACGTTGCGCAAACTATTAACTGGCGAACTACTTACTCTAGCTTCCCGGCAACAATTAATAGACTGGATGGAGGCGGATAAAGTTGCAGGACCACTTCTGCGCTCGGCCCTTCCGGCTGGCTGGTTTATTGCTGATAAATCTGGAGCCGGTGAGCGTGGGTCTCGCGGTATCATTGCAGCACTGGGGCCAGATGGTAAGCCCTCCCGTATCGTAGTTATCTACACGACGGGGAGTCAGGCAACTATGGATGAACGAAATAGACAGATCGCTGAGATAGGTGCCTCACTGATTAAGCATTGGTAACTGTCAGACCAAGTTTACTCATATATACTTTAGATTGATTTAAAACTTCATTTTTAATTTAAAAGGATCTAGGTGAAGATCCTTTTTGATAATCTCATGACCAAAATCCCTTAACGTGAGTTTTCGTTCCACTGAGCGTCAGACCCCGTAGAAAAGATCAAAGGATCTTCTTGAGATCCTTTTTTTCTGCGCGTAATCTGCTGCTTGCAAACAAAAAAACCACCGCTACCAGCGGTGGTTTGTTTGCCGGATCAAGAGCTACCAACTCTTTTTCCGAAGGTAACTGGCTTCAGCAGAGCGCAGATACCAAATACTGTCCTTCTAGTGTAGCCGTAGTTAGGCCACCACTTCAAGAACTCTGTAGCACCGCCTACATACCTCGCTCTGCTAATCCTGTTACCAGTGGCTGCTGCCAGTGGCGATAAGTCGTGTCTTACCGGGTTGGACTCAAGACGATAGTTACCGGATAAGGCGCAGCGGTCGGGCTGAACGGGGGGTTCGTGCACACAGCCCAGCTTGGAGCGAACGACCTACACCGAACTGAGATACCTACAGCGTGAGCTATGAGAAAGCGCCACGCTTCCCGAAGGGAGAAAGGCGGACAGGTATCCGGTAAGCGGCAGGGTCGGAACAGGAGAGCGCACGAGGGAGCTTCCAGGGGGAAACGCCTGGTATCTTTATAGTCCTGTCGGGTTTCGCCACCTCTGACTTGAGCGTCGATTTTTGTGATGCTCGTCAGGGGGGCGGAGCCTATGGAAAAACGCCAGCAACGCGGCCTTTTTACGGTTCCTGGCCTTTTGCTGGCCTTTTGCTCACATGTTCTTTCCTGCGTTATCCCCTGATTCTGTGGATAACCGTATTACCGCCTTTGAGTGAGCTGATACCGCTCGCCGCAGCCGAACGACCGAGCGCAGCGAGTCAGTGAGCGAGGAAGCGGAAGAGCGCCTGATGCGGTATTTTCTCCTTACGCATCTGTGCGGTATTTCACACCGCATATGGTGCACTCTCAGTACAATCTGCTCTGATGCCGCATAGTTAAGCCAGTATACACTCCGCTATCGCTACGTGACTGGGTCATGGCTGCGCCCCGACACCCGCCAACACCCGCTGACGCGCCCTGACGGGCTTGTCTGCTCCCGGCATCCGCTTACAGACAAGCTGTGACCGTCTCCGGGAGCTGCATGTGTCAGAGGTTTTCACCGTCATCACCGAAACGCGCGAGGCAGCTGCGGTAAAGCTCATCAGCGTGGTCGTGAAGCGATTCACAGATGTCTGCCTGTTCATCCGCGTCCAGCTCGTTGAGTTTCTCCAGAAGCGTTAATGTCTGGCTTCTGATAAAGCGGGCCATGTTAAGGGCGGTTTTTTCCTGTTTGGTCACTGATGCCTCCGTGTAAGGGGGATTTCTGTTCATGGGGGTAATGATACCGATGAAACGAGAGAGGATGCTCACGATACGGGTTACTGATGATGAACATGCCCGGTTACTGGAACGG

Supplementary References:

1. Noyes, M.B., Meng, X., Wakabayashi, A., Sinha, S., Brodsky, M.H. and Wolfe, S.A. (2008) A systematic characterization of factors that regulate Drosophila segmentation via a bacterial one-hybrid system. *Nucleic Acids Res*, **36**, 2547-2560.

2. Meng, X., Noyes, M.B., Zhu, L.J., Lawson, N.D. and Wolfe, S.A. (2008) Targeted gene inactivation in zebrafish using engineered zinc-finger nucleases. *Nat Biotechnol*, **26**, 695-701.

3. Noyes, M.B. (2012) Analysis of specific protein-DNA interactions by bacterial one-hybrid assay. *Methods Mol Biol*, **786**, 79-95.

4. Gupta, A., Christensen, R.G., Rayla, A.L., Lakshmanan, A., Stormo, G.D. and Wolfe, S.A. (2012) An optimized two-finger archive for ZFN-mediated gene targeting. *Nat Methods*, **9**, 588-590.

5. Noyes, M.B., Christensen, R.G., Wakabayashi, A., Stormo, G.D., Brodsky, M.H. and Wolfe, S.A. (2008) Analysis of homeodomain specificities allows the family-wide prediction of preferred recognition sites. *Cell*, **133**, 1277-1289.
